# The relationship between preserved ratio impaired spirometry and mortality in the myocardial infarction survivors: a population-based cohort study

**DOI:** 10.1186/s12872-023-03352-2

**Published:** 2023-06-29

**Authors:** Dan Li, Zhishen Ruan, Shen Xie, Shunchao Xuan, Hengyi Zhao, Bo Wu

**Affiliations:** 1 The First Clinical College, Shandong Chinese Medical University, Ji Nan, People’s Republic of China; 2 Department of Cardiovascular Medicine, The First Affiliated Hospital of Shandong University of Traditional Chinese Medicine, Jinan, People’s Republic of China

**Keywords:** Preserved ratio impaired spirometry, Myocardial infarction, Mortality, Cohort study, Lung function

## Abstract

**Introduction:**

Preserved ratio impaired spirometry (PRISm) is a subtype of pulmonary function abnormality which is characterized by a proportional reduction in non-obstructive expiratory lung volume. Currently, no studies have shown a relationship between PRISm and mortality in myocardial infarction (MI) survivors.

**Methods:**

We used cohort data from U.S. adults who attended the National Health and Nutrition Examination Survey (NHANES) from 2007 to 2012. According to the ratio of forced expiratory volume in the first second (FEV_1_) to forced vital capacity (FVC), we divided lung function into normal spirometry (FEV_1_/ FVC) ≥ 70%, FEV_1_ ≥ 80%), PRISm (FEV_1_/FVC ≥ 70%, FEV_1_ < 80%) and obstructive spirometry (FEV_1_/FVC < 70%). Cox regression was used to estimate the correlation between lung functions and mortality among MI patients. Kaplan-Meier survival curves compared the prognosis of MI with three different lung functions. We further verify the stability of the results by sensitivity analysis.

**Results:**

411 subjects were included in our research. The mean follow-up time for the study was 105 months. Compared with normal spirometry, PRISm was significantly correlated with a greater relative risk for all-cause mortality (adjust HR 3.41, 95% confidence interval [95%CI]: 1.76–6.60, *P* < 0.001) and cardiovascular mortality (adjust HR 13.9, 95%CI: 2.60–74.6, *P* = 0.002). PRISm remains more correlated with all-cause mortality (adjust HR 2.73, 95%CI: 1.28–5.83, *P* = 0.009) relative to obstructive spirometry. The results are basically stable after sensitivity analysis. Kaplan-Meier survival curves showed that patients with PRISm tended to have the lowest survival during the follow-up period.

**Conclusion:**

PRISm is an independent risk factor for all-cause and cardiovascular mortality in MI survivors. The presence of PRISm was associated with a significantly higher risk of all-cause mortality compared with obstructive spirometry.

**Supplementary Information:**

The online version contains supplementary material available at 10.1186/s12872-023-03352-2.

## Introduction

With the renewed therapeutic and management modality, the mortality with myocardial infarction (MI) has decreased considerably for patients in the past two decades [1]. Therefore, effectively improving the remaining quality of life of myocardial infarction (MI) survivors becomes a valuable direction. The exploration of relevant risk factors has a positive effect on the prolongation of life.

As well known, reduced lung function is a significant predictor of cardiovascular disease mortality [2, 3]. Forced expiratory volume in the first second (FEV_1_) / forced vital capacity (FVC) is an important indicator of lung function. Spirometry results can be divided into normal lung function, airway obstruction, and Preserved Ratio Impaired Spirometry (PRISm) [4]. GOLD 2023 provides the first clear definition of PRISm as FEV_1_/FVC greater than or equal to 70% and FEV_1_ less than 80% predicted; GOLD states that not all people have PRISm will eventually develop fixed airflow obstruction, but they should be recognized as “patients” [5]. PRISm has an estimated worldwide prevalence of 6 to 20 percent [6–8]. Recent prospective studies have shown that the presence of PRISm at baseline was significantly associated with cardiovascular and all-cause mortality in the general population [8–11]. Studies show that PRISm is associated with the risk of future infarction [3, 12].

However, no studies have yet correlated PRISm with the prognosis of death in MI survivors. Survivors of MI are ignored. We plan to explore the relationship between PRISm and mortality in the MI population by extracting data from the National Health and Nutrition Examination Survey (NHANES).

## Methods

### Study population

NHANES databases are collected by the United States National Center for Health Statistics and Centers for Disease Control and Prevention based on the entire U.S. population. Survey methodology and sampling design make the statistics nationally representative. The data collection includes a standardized home interview, physical examination and biological specimen. NHANES database was approved by the National Center for Health Statistics Research Ethics Review Board, and all patients had signed informed consent. Our study conforms to the criteria for Strengthening Observational Studies in Epidemiology [13]. All statistics used in the study are available at https://www.cdc.gov/nchs/nhanes.

We collected and analyzed participants recorded through NHANES from 2007 to 2012. Participants received respiratory symptom questionnaires and performed standardized spirometry during this period. The research included 411 adult participants with a history of myocardial infarction. The medical history of MI was based on affirmative answers to the following question from the self-report. “Has a doctor or other health professional ever told you that you/he/she had a heart attack (also called myocardial infarction)?“.

**Fig. 1:**
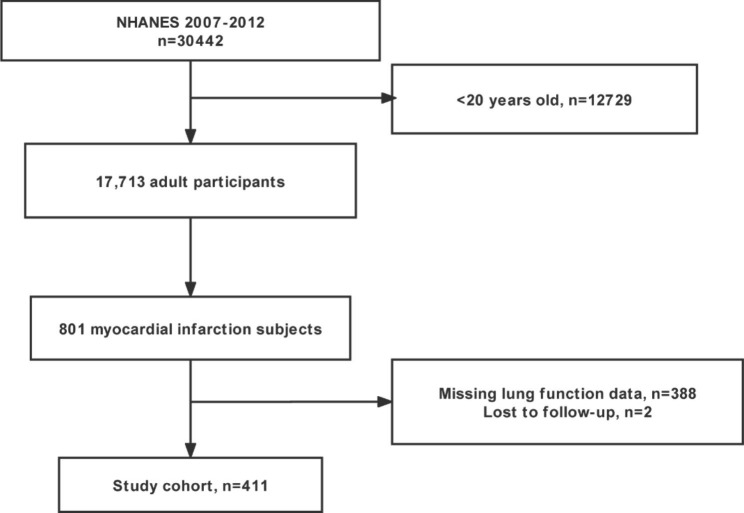
Flow chart

Figure 1 illustrates the detailed inclusion and exclusion criteria.

### Lung function measurements

Patients underwent spirometry after completing the respiratory questionnaire. According to American Thoracic Society (ATS) guidelines, Ohio 822/827 dry-rolling volume seal, water seal or flow-sensing spirometers are used for pre-bronchodilator spirometry [14]. Spirometry is graded according to the ATS quality standards, using only manoeuvres with a quality grade greater than C [15].

Normal spirometry was defined as FEV_1_/FVC greater than or equal to 70% and an FEV_1_ greater than or equal to 80% of the predicted value. Obstructive spirometry was defined as FEV_1_/FVC less than 70%. PRISm was defined as FEV_1_/ FVC greater than or equal to 70% and FEV_1_ less than 80% predicted [16].

### Study covariates

We incorporated demographic data (age, gender, race, education, body mass index [BMI] and smoking status), which were derived from self-reported. The race was divided into Non-Hispanic White, Mexican American, Non-Hispanic Black and other races. BMI was classified into 3 levels (< 25, 25–30 and > 30 kg/m^2^). Considering the relationship between smoking and lung health, we categorized smoking status as never (smoking less than 100 cigarettes in a lifetime), previous (smokes more than 100 cigarettes but has quit), and current.

In addition, we included diabetes, hypertension, chronic kidney disease, heart failure, and stroke as comorbidities. Diabetes was defined as the presence of one of the following conditions (diagnosed by a physician, taking glucose-lowering medication, glycosylated hemoglobin ≥ 6.5%, fasting blood-glucose ≥ 7.0 mmol/L, glucose tolerance test ≥ 11.1 mmol/L). Hypertension was defined as the presence of one of the following conditions (diagnosed by a physician, using antihypertensive medications, systolic blood pressure ≥ 140 mmHg or diastolic blood pressure ≥ 90 mmHg). Chronic kidney disease (CKD) was considered present if the glomerular filtration rate (GFR) < 60 ml/min/1.73 m [2] or urinary albumin-creatinine ratio (UACR) ≥ 30 mg/g [17]. The medical history of heart failure and stroke was derived from the patient’s self-report [18]. People with heart failure/stroke were defined as those who answered “yes” to the following questions: “Have you ever been told you have heart failure/stroke?“ [19].

The information on substance use (antiplatelet, statin and β-blocker) obtained from home interviews was also considered as covariates. This reflected the prescription drug utilization of the patient within the last 30 days.

### Study outcomes

All-cause mortality and cardiovascular mortality in the MI population during follow-up were the primary outcomes of the study. NHANES-linked National Death Index public access files determined mortality distribution status and cause of death statistics. According to the International Statistical Classification of Diseases 10th Revision (ICD-10) codes, cardiovascular mortality is defined as death caused by acute rheumatic fever and chronic rheumatic heart diseases (I00-I09), hypertensive heart disease (I11), hypertensive heart and renal diseases (I13), ischemic heart disease (I20-I25) and other heart diseases (I26-I51), including atrial fibrillation (I48) and heart failure (I50) [20].

### Statistical analysis

Descriptive statistics are weighted based on demographic data. Weighted cox regression was used to estimate the correlation between different lung functions (normal spirometry, PRISm and obstructive spirometry) and mortality among MI patients. Furthermore, we adjusted for potential bias using three models. Model 1 adjusted nothing, model 2 adjusted age, gender and race, and model 3 adjusted for model 2 plus education, BMI, smoking, diabetes, hypertension, CKD, heart failure, stroke, use of antiplatelet, use of statin, and use of β-blocker. Kaplan-Meier survival curves were used to assess the influence of three lung functions on all-cause and cardiovascular mortality in MI survivors. To further verify the stability of the results, we performed a sensitivity analysis using propensity score matching (PSM). After the three lung function groups were matched two-by-two separately, regression analysis was conducted to verify the relationship between lung function and mortality. Statistical significance was assessed at a two-sided P value < 0.05. Since the missing values of all covariates were less than 0.5%, we ignored them in the analysis.

The statistical analyses were performed with the statistical software packages and Free Statistics software versions (1.5) and R v4.1.3 (http://www.R-project.org, The R Foundation).

## Results

### Baseline characteristics

411 patients with MI were enrolled in our study. The baseline characteristics of the study population are shown in Table 1, with a weighted population of 411 participants reflecting 4,258,261 Americans. The mean age of subjects was 62.3. Most subjects were male (68.9%) and Non-Hispanic White (53.8%). 48.7% of the subjects had a BMI greater than 30 kg/m^2^. Former and current smokers accounted for 68.1% of MI survivors. Among the MI participants, 45.9% suffered from diabetes, and 36.0% from CKD. 23.1% of patients on antiplatelet drugs. Regarding mortality, 31.5% of subjects died during a mean follow-up of 105 months, of which 9.7% died of cardiovascular disease. Patients with obstructive pulmonary function were older, while patients with PRISm had a higher BMI. Patients with abnormal spirometry (including PRISm and obstructive pulmonary) tend to have a smoking history, are prone to combined diabetes and CKD, and have a high mortality rate.

**Table 1:** Characteristics of participants, 2007–2012 NHANES (n = 411)

Characteristics	Total (N = 411)	Normal spirometry (N = 206)	PRISm (N = 71)	Obstructive spirometry (N = 134)	*P*
Age, years	62.3 ± 11.2	60.0 ± 12.0	60.0 ± 10.6	67.1 ± 8.6	< 0.001
Male, n (%)	283 (68.9)	138 (67)	44 (62)	101 (75.4)	0.102
Race/ethnicity, n (%)					< 0.001
Non-Hispanic White	221 (53.8)	93 (45.1)	32 (45.1)	96 (71.6)	
Mexican American	46 (11.2)	32 (15.5)	7 (9.9)	7 (5.2)	
Non-Hispanic Black	87 (21.2)	54 (26.2)	16 (22.5)	17 (12.7)	
Other Race	57 (13.9)	27 (13.1)	16 (22.5)	14 (10.4)	
Education, n (%)					0.011
< High school diploma	71 (17.3)	36 (17.5)	12 (16.9)	23 (17.2)	
Completed high school	188 (45.7)	78 (37.9)	41 (57.7)	69 (51.5)	
≥ College	152 (37.0)	92 (44.7)	18 (25.4)	42 (31.3)	
BMI, kg/m^2^, %					0.001
< 25	84 (20.4)	37 (18)	10 (14.1)	37 (27.6)	
25–30	127 (30.9)	59 (28.6)	17 (23.9)	51 (38.1)	
> 30	200 (48.7)	110 (53.4)	44 (62)	46 (34.3)	
Smoke, %					< 0.001
Never smoker	131 (31.9)	89 (43.2)	20 (28.2)	22 (16.4)	
Former smoker	163 (39.7)	70 (34)	25 (35.2)	68 (50.7)	
Current smoker	117 (28.5)	47 (22.8)	26 (36.6)	44 (32.8)	
Comorbidities, %					
Hypertension	313 (76.2)	163 (79.1)	49 (69)	101 (75.4)	0.219
Diabetes	188 (45.9)	77 (37.6)	47 (66.2)	64 (47.8)	< 0.001
CKD	143 (36.0)	60 (30)	28 (41.8)	55 (42.3)	0.042
Heart failure	114 (28.3)	54 (26.6)	22 (31)	38 (29.5)	0.731
Stroke	70 (17.1)	34 (16.6)	16 (22.5)	20 (14.9)	0.374
Medication use, %					
Antiplatelet	95 (23.1)	50 (24.3)	13 (18.3)	38 (28.4)	0.01
Statin	226 (55.1)	106 (51.5)	36 (50.7)	84 (63.2)	0.076
β-blocker	233 (56.8)	111 (53.9)	40 (56.3)	82 (61.7)	0.368
Mortality					
All-cause	129 (31.5)	41 (19.9)	31 (44.3)	57 (42.5)	< 0.001
Cardiovascular-related	40 (9.7)	11 (5.3)	11 (15.5)	18 (13.4)	0.01

BMI: body mass index, CKD: chronic kidney diseases, PRISm: preserved ratio impaired spirometry

### Association between lung functions and mortality

**Fig. 2:**
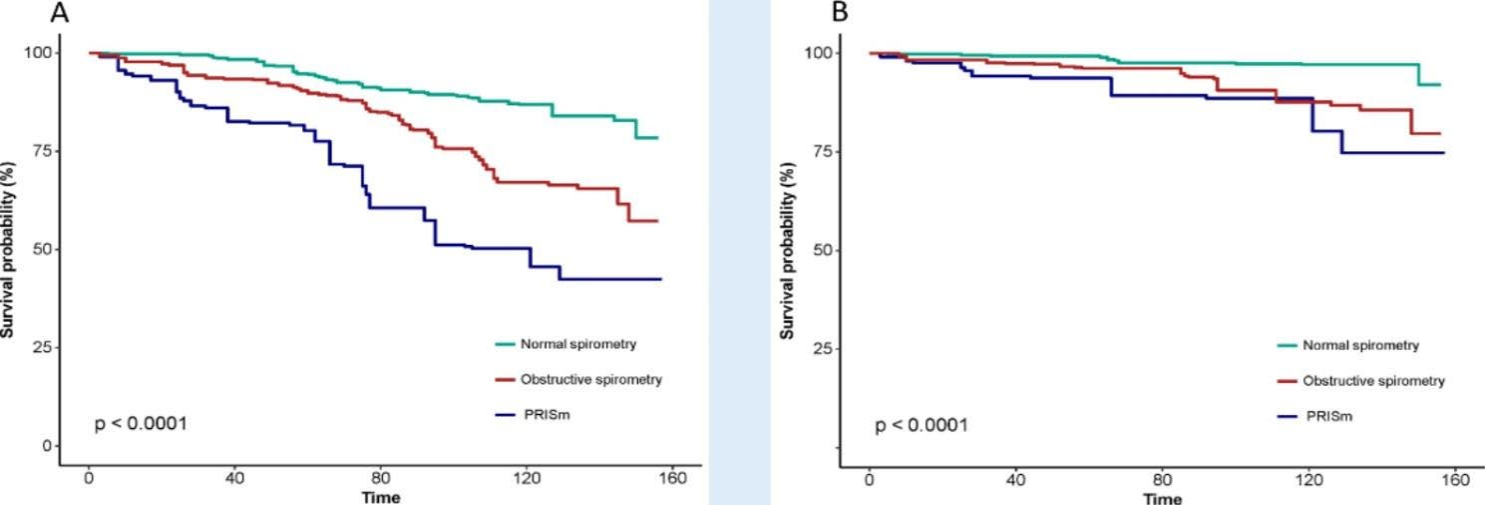
Kaplan-Meier survival curves between different lung functions and mortality in MI survivors **(A)** All-cause mortality, **(B)** Cardiovascular mortality

Table 2 shows the correlation of different lung functions with all-cause mortality and cardiovascular mortality. Compared with normal and obstructive spirometry, the adjusted HRs for all-cause mortality for PRISm were 3.41 (95% confidence interval [95%CI]: 1.76–6.60, *P* < 0.001) and 2.73 (95%CI: 1.28–5.83, *P* = 0.009), respectively. It can be seen that PRISm was an 173% higher risk of death than obstructive spirometry in terms of all-cause mortality. At baseline with normal spirometry, we found that PRISm (HR: 13.9, 95%CI: 2.60–74.6, *P* = 0.002) and obstructive spirometry (HR: 2.02, 95%CI: 1.08–3.80, *P* = 0.028) were significantly associated with a greater risk of cardiovascular mortality. Kaplan-Meier survival curves (Fig. 2) showed that PRISm patients had the lowest survival rates during follow-up.

**Table 2:** Weighted relationship between lung function and mortality in patients with myocardial infarction

		Model 1 h (95% CI)		*P*		Model 2 h (95% CI)		*P*		Model 3 h (95% CI)	*P*	
**All-cause mortality**												
Normal spirometry	1(Ref)				1(Ref)				1(Ref)			
PRISm	5.03 (3.03–8.34)		< 0.001		4.76 (2.98–7.58)		< 0.001		3.41 (1.76–6.60)		< 0.001	
Obstructive spirometry	2.48 (1.40–4.41)		< 0.001		1.66 (0.93–2.96)		0.09		1.16 (0.85–1.58)		0.34	
PRISm vs. Obstructive spirometry	2.03 (1.13–3.64)		0.018		2.87 (1.57–5.26)		< 0.001		2.73 (1.28–5.83)		0.009	
**Cardiovascular mortality**												
Normal spirometry	1(Ref)				1(Ref)				1(Ref)			
PRISm	6.74 (2.27-20.0)		< 0.001		6.52 (2.07–20.55)		0.001		13.9 (2.60–74.6)		0.002	
Obstructive spirometry	4.29 (1.71–10.8)		0.002		3.22 (1.34–7.72)		0.009		2.02 (1.08–3.80)		0.028	
PRISm vs. Obstructive spirometry	1.57 (0.59–4.18)		0.37		2.03 (0.68–6.06)		0.21		2.62 (0.35–19.5)		0.35	

Abbreviations: PRISm, preserved ratio impaired spirometry

Notes: Model 1 adjusted nothing, model 2 was adjusted for age, gender, and race, and model 3 was adjusted for model 2 plus education, BMI, smoking, diabetes, hypertension, CKD, heart failure, stroke, use of antiplatelet, use of statin, and use of β-blocker

### Sensitivity analysis

To further verify the stability of the results, we performed PSM of the three pulmonary function groups. The three groups were matched two by two (Table S1-S3). None of the differences in baseline information after matching were statistically significant (*P* > 0.05), indicating significant overlap in propensity scores. The post-matching COX regressions indicated that the main results were essentially the same as before PSM (Table S4). The trend towards an increased risk of all-cause mortality for PRISm compared to obstructive spirometry persisted, however the p-value was 0.083.

## Discussion

To our knowledge, this is the first study to examine the relationship between PRISm and mortality in MI survivors. As shown in Table 2, for all-cause and cardiovascular mortality, the presence of PRISm disease at baseline was significantly associated with an increased absolute risk relative to normal spirometry. In addition, the all-cause mortality rate for PRISm was even higher than that for obstructive disease. Although the result after PSM was negative, there was still a trend towards higher mortality risk in the PRISm group. The reduced sample size after matching contributes to explain it. Sensitivity analysis demonstrated that the other results were generally stable. Limited data suggest that 50% of patients with PRISm may return to normal spirometry levels [11]. Therefore, dynamic monitoring of pulmonary function in patients with MI in the presence of PRISm has positive implications for assessing their prognosis. Unfortunately, this study was unable to achieve dynamic detection of lung function, which is to be supplemented by subsequent studies.

The prevalence of hypertension and diabetes is higher in patients with abnormal spirometry [21, 22], and our experiments verify that this finding is also generalized in patients with MI (Table 1). The current explanation for this phenomenon is a combination of pulmonary inflammation, chronic systemic inflammation and oxidative stress [23]. Fibrinogen and other inflammation-sensitive plasma proteins (ISPs) are components of the inflammatory response and there is an increased incidence of MI in those with high plasma protein levels [24, 25]. Other inflammatory markers, such as C-reactive protein, fibronectin, pro-inflammatory cytokines and leukocyte levels, are further increased when lung function decreases [26–28]. Researchers consider that inflammatory cells and pro-inflammatory mediators in the lungs spill out of the systemic circulation, leading to chronic systemic inflammation [23, 29]. In addition, obesity, dyslipidemia and metabolic syndrome were also found to be independently associated with PRISm [30, 31]. This explains the complications of PRISm to a certain extent.

Although obstructive breathing patterns are more common, restrictive breathing patterns have shown a stronger association with cardiovascular disease, according to research findings [32]. The complex interactions between airflow obstruction, pulmonary vascular remodeling, and cardiac function are not well understood. Numerous studies have shown an association between reduced lung function and future cardiovascular events (atrial fibrillation, acute coronary syndrome and heart failure) [33–35]. Ramalho et al. [36] suggested that a FVC decrease with or without FEV_1_ ratio decreasemay represent different pathological processes, such as LV underfilling observed in the obstructive type and LV diastolic dysfunction observed in the restrictive type. This may explain the difference in prognosis between PRISm and obstructive lung disease in MI.

The strength of our report is the use of two indicators, FEV_1_ and FVC, to classify subjects into normal spirometry, obstructive spirometry, and PRISm. Previous studies have analyzed FEV_1_ and FVC as continuous variables [3, 37], which may have resulted in omission of the PRISm population. In addition, by means of complex sampling weighting, the sample for this study is representative of the entire U.S. population data. Undeniably, there are some limitations in our study. First, confounding factors may create potential bias. Second, since the presence of disease was obtained through self-report, it could lead to potential bias. However, the sensitivity of self-reported MI ranged from 89.0 to 97.7% and the specificity from 90–99% [38–40]. Several studies have suggested that self-reported MI can be reliably applied to include and exclude patients at baseline [41, 42]. Finally, although the study encompassed ethnic diversity, participants were confined to the United States and the findings cannot be generalized outside the United States.

## Conclusions

PRISm is an independent risk factor for all-cause and cardiovascular mortality in MI survivors. The presence of PRISm was associated with a significantly higher risk of all-cause mortality compared with obstructive spirometry. This provides new insight into the link between lung function and MI.

## Electronic supplementary material

Below is the link to the electronic supplementary material.

Supplementary file: Sensitivity analysis

## Data Availability

Data in the article can be obtained from the NHANES database (https://www.cdc.gov/nchs/nhanes/index.htm).
