# Supplementary material for: The relationship between preserved ratio impaired spirometry and mortality in the myocardial infarction survivors: a population-based cohort study
Source: BMC Cardiovasc Disord. 2023 Jun 29;23:331. doi: 10.1186/s12872-023-03352-2 (PMC10311719; doi:10.1186/s12872-023-03352-2)
Supplement: Supplementary file 1 — Supplementary file: Sensitivity analysis [file 12872_2023_3352_MOESM1_ESM.docx]

Table S1 The characteristics of the population between Normal spirometry and PRISM after PSM (n=116)

| **Variables** | **Total**  **(N=116)** | **Normal spirometry**  **(N=58)** | **PRISm**  **(N=58)** | ***P*** |
| --- | --- | --- | --- | --- |
| Age, years | 60.2 ± 11.5 | 60.4 ± 12.2 | 59.9 ± 10.9 | 0.798 |
| Male, n (%) | 77 (66.4) | 40 (69) | 37 (63.8) | 0.555 |
| Race/ethnicity, n (%) |  |  |  | 0.808 |
| Non-Hispanic White | 51 (44.0) | 26 (44.8) | 25 (43.1) |  |
| Mexican American | 11 (09.5) | 4 (6.9) | 7 (12.1) |  |
| Non-Hispanic Black | 30 (25.9) | 16 (27.6) | 14 (24.1) |  |
| Other Race | 24 (20.7) | 12 (20.7) | 12 (20.7) |  |
| Education, n (%) |  |  |  | 0.977 |
| < High school diploma | 18 (15.5) | 9 (15.5) | 9 (15.5) |  |
| Completed high school | 65 (56.0) | 33 (56.9) | 32 (55.2) |  |
| ≥ College | 33 (28.4) | 16 (27.6) | 17 (29.3) |  |
| BMI, kg/m^2^, % |  |  |  | 0.900 |
| < 25 | 19 (16.4) | 10 (17.2) | 9 (15.5) |  |
| 25-30 | 28 (24.1) | 13 (22.4) | 15 (25.9) |  |
| > 30 | 69 (59.5) | 35 (60.3) | 34 (58.6) |  |
| Smoke, % |  |  |  | 0.621 |
| Never smoker | 33 (28.4) | 15 (25.9) | 18 (31) |  |
| Former smoker | 43 (37.1) | 24 (41.4) | 19 (32.8) |  |
| Current smoker | 40 (34.5) | 19 (32.8) | 21 (36.2) |  |
| Comorbidities, % |  |  |  |  |
| Hypertension | 87 (75.0) | 43 (74.1) | 44 (75.9) | 0.830 |
| Diabetes | 67 (57.8) | 33 (56.9) | 34 (58.6) | 0.851 |
| CKD | 45 (38.8) | 25 (43.1) | 20 (34.5) | 0.341 |
| Heart failure | 39 (33.6) | 19 (32.8) | 20 (34.5) | 0.844 |
| Stroke | 24 (20.7) | 11 (19) | 13 (22.4) | 0.647 |
| Medication use, % |  |  |  |  |
| Antiplatelet | 24 (20.7) | 13 (22.4) | 11 (19) | 0.647 |
| Statin | 61 (52.6) | 30 (51.7) | 31 (53.4) | 0.852 |
| β-blocker | 62 (53.4) | 31 (53.4) | 31 (53.4) | 1.000 |

BMI: body mass index, CKD: chronic kidney diseases, PRISm: preserved ratio impaired spirometry, PSM: propensity score matching.

| **Variables** | **Total**  **(N=168)** | **Normal spirometry**  **(N = 84)** | **Obstructive spirometry**  **(N=84)** | ***P*** |
| --- | --- | --- | --- | --- |
| Age, years | 64.4 ± 10.3 | 63.6 ± 11.5 | 65.1 ± 9.0 | 0.374 |
| Male, n (%) | 134 (79.8) | 68 (81) | 66 (78.6) | 0.701 |
| Race/ethnicity, n (%) |  |  |  | 0.762 |
| Non-Hispanic White | 108 (64.3) | 54 (64.3) | 54 (64.3) |  |
| Mexican American | 13 (7.7) | 6 (7.1) | 7 (8.3) |  |
| Non-Hispanic Black | 25 (14.9) | 11 (13.1) | 14 (16.7) |  |
| Other Race | 22 (13.1) | 13 (15.5) | 9 (10.7) |  |
| Education, n (%) |  |  |  | 0.832 |
| < High school diploma | 31 (18.5) | 17 (20.2) | 14 (16.7) |  |
| Completed high school | 75 (44.6) | 37 (44) | 38 (45.2) |  |
| ≥ College | 62 (36.9) | 30 (35.7) | 32 (38.1) |  |
| BMI, kg/m^2^, % |  |  |  | 0.735 |
| < 25 | 36 (21.4) | 20 (23.8) | 16 (19) |  |
| 25-30 | 57 (33.9) | 27 (32.1) | 30 (35.7) |  |
| > 30 | 75 (44.6) | 37 (44) | 38 (45.2) |  |
| Smoke, % |  |  |  | 0.429 |
| Never smoker | 42 (25.0) | 21 (25) | 21 (25) |  |
| Former smoker | 81 (48.2) | 37 (44) | 44 (52.4) |  |
| Current smoker | 45 (26.8) | 26 (31) | 19 (22.6) |  |
| Comorbidities, % |  |  |  |  |
| Hypertension | 128 (76.2) | 63 (75) | 65 (77.4) | 0.717 |
| Diabetes | 65 (38.7) | 32 (38.1) | 33 (39.3) | 0.874 |
| CKD | 57 (33.9) | 29 (34.5) | 28 (33.3) | 0.871 |
| Heart failure | 45 (26.8) | 23 (27.4) | 22 (26.2) | 0.862 |
| Stroke | 28 (16.7) | 16 (19) | 12 (14.3) | 0.408 |
| Medication use, % |  |  |  |  |
| Antiplatelet | 47 (28.0) | 24 (28.6) | 23 (27.4) | 0.864 |
| Statin | 102 (60.7) | 53 (63.1) | 49 (58.3) | 0.527 |
| β-blocker | 105 (62.5) | 52 (61.9) | 53 (63.1) | 0.873 |

Table S2 The characteristics of the population between Normal spirometry and Obstructive spirometry after PSM (n=168)

BMI: body mass index, CKD: chronic kidney diseases, PSM: propensity score matching.

| **Variables** | **Total**  **(N=96)** | **PRISm**  **(N = 48)** | **Obstructive spirometry**  **(N=48)** | ***P*** |
| --- | --- | --- | --- | --- |
| Age, years | 62.9 ± 8.9 | 63.2 ± 8.2 | 62.7 ± 9.7 | 0.794 |
| Male, n (%) | 60 (62.5) | 31 (64.6) | 29 (60.4) | 0.673 |
| Race/ethnicity, n (%) |  |  |  | 0.657 |
| Non-Hispanic White | 51 (53.1) | 25 (52.1) | 26 (54.2) |  |
| Mexican American | 10 (10.4) | 4 (8.3) | 6 (12.5) |  |
| Non-Hispanic Black | 21 (21.9) | 10 (20.8) | 11 (22.9) |  |
| Other Race | 14 (14.6) | 9 (18.8) | 5 (10.4) |  |
| Education, n (%) |  |  |  | 0.639 |
| < High school diploma | 20 (20.8) | 9 (18.8) | 11 (22.9) |  |
| Completed high school | 50 (52.1) | 24 (50) | 26 (54.2) |  |
| ≥ College | 26 (27.1) | 15 (31.2) | 11 (22.9) |  |
| BMI, kg/m^2^, % |  |  |  | 0.952 |
| < 25 | 17 (17.7) | 8 (16.7) | 9 (18.8) |  |
| 25-30 | 25 (26.0) | 13 (27.1) | 12 (25) |  |
| > 30 | 54 (56.2) | 27 (56.2) | 27 (56.2) |  |
| Smoke, % |  |  |  | 0.708 |
| Never smoker | 19 (19.8) | 10 (20.8) | 9 (18.8) |  |
| Former smoker | 35 (36.5) | 19 (39.6) | 16 (33.3) |  |
| Current smoker | 42 (43.8) | 19 (39.6) | 23 (47.9) |  |
| Comorbidities, % |  |  |  |  |
| Hypertension | 69 (71.9) | 33 (68.8) | 36 (75) | 0.496 |
| Diabetes | 62 (64.6) | 31 (64.6) | 31 (64.6) | 1.000 |
| CKD | 37 (38.5) | 19 (39.6) | 18 (37.5) | 0.834 |
| Heart failure | 26 (27.1) | 13 (27.1) | 13 (27.1) | 1.000 |
| Stroke | 18 (18.8) | 9 (18.8) | 9 (18.8) | 1.000 |
| Medication use, % |  |  |  |  |
| Antiplatelet | 19 (19.8) | 8 (16.7) | 11 (22.9) | 0.442 |
| Statin | 50 (52.1) | 25 (52.1) | 25 (52.1) | 1.000 |
| β-blocker | 54 (56.2) | 27 (56.2) | 27 (56.2) | 1.000 |

Table S3 The characteristics of the population between PRISm and Obstructive spirometry after PSM (n=96)

BMI: body mass index, CKD: chronic kidney diseases, PRISm: preserved ratio impaired spirometry, PSM: propensity score matching.

Table S4 Weighted relationship between lung function and mortality in patients with myocardial infarction after PSM.

|  | **HR (95% CI)** | ***P*** |
| --- | --- | --- |
| **All-cause mortality** |  |  |
| Normal spirometry | 1(Ref) |  |
| PRISm | 4.76 (2.06-11.0) | <0.001 |
| Obstructive spirometry | 1.19 (0.87-1.64) | 0.28 |
| PRISm vs  Obstructive spirometry | 2.20 (0.90-5.36) | 0.083 |
| **Cardiovascular mortality** |  |  |
| Normal spirometry | 1(Ref) |  |
| PRISm | 15.3 (1.17-199) | 0.038 |
| Obstructive spirometry | 1.73 (1.01-2.99) | 0.048 |
| PRISm vs  Obstructive spirometry | 2.54 (0.60-10.68) | 0.20 |

Notes: Adjusted for age, gender, and race, education, BMI, smoking, diabetes, hypertension, CKD, heart failure, stroke, use of antiplatelet, use of statin, and use of β-blocker.

Abbreviations: BMI, body mass index; CKD, chronic kidney diseases; PRISm, preserved ratio impaired spirometry; PSM, propensity score matching.
